# Whole brain volume loss is associated with a short-term disability progression in relapse-activity free multiple sclerosis

**DOI:** 10.1007/s00415-025-13343-2

**Published:** 2025-10-21

**Authors:** Roland Opfer, Lothar Spies, Julia Krüger, Thomas Buddenkotte, Holger Roick, Manda Jankovic, Nicolaj Witt, Sylke Domke, Ralf Kubalek, Gerd Reifschneider, Jürgen Kunz, Ilias Nastos, Felicita Heidler, George Trendelenburg, Andreas Stockert, Deborah K. Erhart, Hayrettin Tumani, Hagen H. Kitzler, Tjalf Ziemssen

**Affiliations:** 1 jung diagnostics GmbH, Hamburg, Germany; 2 UKE Department of Diagnostic and Interventional Radiology and Nuclear Medicine, University Medical Center Hamburg- Eppendorf, Hamburg, Germany; 3 E/M/S/A, Singen, Germany; 4 MS-Spezialambulanz, Sauerlandklinik Hachen, Sundern, Germany; 5 Neurologische Praxis Am Israelitischen Krankenhaus, Hamburg, Germany; 6 Neurologische Praxis Chemnitz-Rabenstein, Chemnitz, Germany; 7 Neuropraxis München Süd, Unterhaching, Germany; 8 Neuro Centrum Odenwald, Erbach, Germany; 9 Neurozentrum Ravensburg, Ravensburg, Germany; 10 MS Zentrum, Bochum, Germany; 11 Ökumenisches Hainich Klinikum gGmbH, Mühlhausen, Germany; 12 Department of Neurology, University Medical Center, Göttingen, Germany; 13 Facharztpraxis Für Neurologie Und Psychiatrie, Pforzheim, Germany; 14 Department of Neurology, University Hospital Ulm, Ulm, Germany; 15 Institute of Diagnostic and Interventional Neuroradiology, University Hospital Carl Gustav Carus, Technical University of Dresden, Dresden, Germany; 16 MS Center, Center of Clinical Neuroscience, Neurological Clinic, University Hospital Carl Gustav Carus, Technical University of Dresden, Dresden, Germany

**Keywords:** Magnetic resonance imaging, Multiple sclerosis, Whole brain volume loss, Disability progression, Brain atrophy

## Abstract

**Background:**

Reliable biomarkers for disability progression independent of relapse activity (PIRA) in multiple sclerosis (MS) applicable in routine patient care are urgently needed. This study reports results from an ongoing multicenter, prospective, observational study with the primary objective of investigating the association of change in brain MRI biomarkers and PIRA.

**Methods:**

In total 453 active relapsing remitting patients from 19 sites with baseline (BL) and one-year follow-up (FU) visits were included. At each visit, medication, relapse activity, and EDSS were recorded. MRI included 3D-T1 and 2D/3D-FLAIR. BL and FU scans enabled extraction of new/enlarged T2-lesions and brain volume loss (BVL) (annualized). Correlations and logistic regression assessed associations between EDSS progression and BVL/year.

**Results:**

At BL an EDSS (25%/50%/75%) of 1.5/2.0/2.5 and a time since first MS diagnosis of 2.1/5.7/11.4 years were observed. Change (FU-BL): ΔEDSS was 0.0/0.0/0.0, BVL/year was $-$0.5/$-$0.3/$-$0.0%, age-adjusted BVL/year was $-$0.4/$-$0.1/0.2%, and number of new/enlarged T2-lesions per year was 0.0/0.0/0.6. 75% of patients were relapse activity-free during the observation period, 72% had no new/enlarged T2-lesions, and 56% were free of both. BVL/year (adjusted for age) correlated with ΔEDSS (*r* = $-$0.14, *p* = 0.002) for all patients, but also for sub-cohorts of patients without new/enlarged T2-lesions and without relapses (*r* =$-$0.17, *p *= 0.008). BVL/year was significantly associated with EDSS progression in the logistic regression model (*p* < 0.001). The risk of EDSS progression increases from 15% to 19% (relative risk increase of 26%), when BVL/year declines from $-$0.5% to $-$1.0%.

**Conclusions:**

BVL over one year was significantly associated with EDSS progression in the absence of relapses or new lesions, confirming its value as a short-term risk marker for disability progression in MS.

## Introduction

Brain atrophy, defined as whole brain volume loss (BVL), measured using longitudinal magnetic resonance imaging (MRI) scans, is an important marker of neurodegeneration in patients with multiple sclerosis (MS). BVL is accelerated in MS patients compared to BVL in normal ageing [42], it occurs early in the disease course [33], and persists throughout the disease [10]. BVL is used as a primary efficacy endpoint in phase II MS drug trials [25]. Modern disease-modifying therapies (DMTs) for MS slow down BVL [15, 26, 44]. A meta-analysis demonstrated that the treatment effect of DMTs on slowing down disability progression is related to their effect on reducing BVL rates, which is independent of their effect on reducing T2-lesion activity on MRI [41]. It is controversial, whether BVL can be used as a marker for disease and therapy monitoring in clinical routine [1, 48]. The latest MAGNIMS consensus statement recommends measuring BVL to better assess the global disease burden [37]. Most of the obstacles mentioned in these articles have now been overcome. There are age-dependent thresholds that allow a differentiation of normal and pathological BVL [2, 31, 42]. In addition, the extend of the expected measurement error and possible short-term biological fluctuations in BVL (caused, for instance, by different hydration status [12]) have been determined [27, 32]. Deep learning (DL)-based methods for estimating BVL have recently been proposed [27, 46] as an alternative to the traditional SIENA method [40]. DL-based approaches have shown greater robustness and higher sensitivity in detecting accelerated BVL compared to SIENA [27, 34]. Critics of BVL for the clinical routine point out [1] that the most important prerequisite for reliable BVL measurement in clinical routine is the use of standardized, high-quality MRI protocols, whereby baseline (BL) and follow-up (FU) scans must be performed on the same scanner and protocol [47]. A large, non-interventional study with 6,718 MS patients from 297 study centers (MS clinics and outpatient facilities), conducted in Germany from 2016–2020, showed that standardized and high-quality MRI protocols are feasible in day-to-day clinical routine. 9,000 standardized MRIs were collected from 183 different imaging centers, centrally analyzed (including BVL measurement) and reports delivered to the study centers. A systematic survey of the participating physicians revealed that 72.1% of participating physicians observed a strong/very strong correlation between the quantitative MRI parameters (including BVL in case of FU MRI) and the clinical picture of the patients [39]. Currently available DMTs greatly reduce the risk of relapses and lesion activity, but have only limited impact on disability progression independent of relapse activity (PIRA) [17, 21]. Imaging and non-imaging biomarkers for PIRA are urgently needed [5]. In this article, we present initial results of a multicenter, prospective, observational, and non-interventional study (German Clinical Trials Register: DRKS00030403) designed to investigate the association between MS-related disability progression and BVL in routine clinical care. The primary objective is to generate prospective evidence to qualify BVL as risk factor or even a predictor for short-term disability progression in MS.

**Fig. 1:**
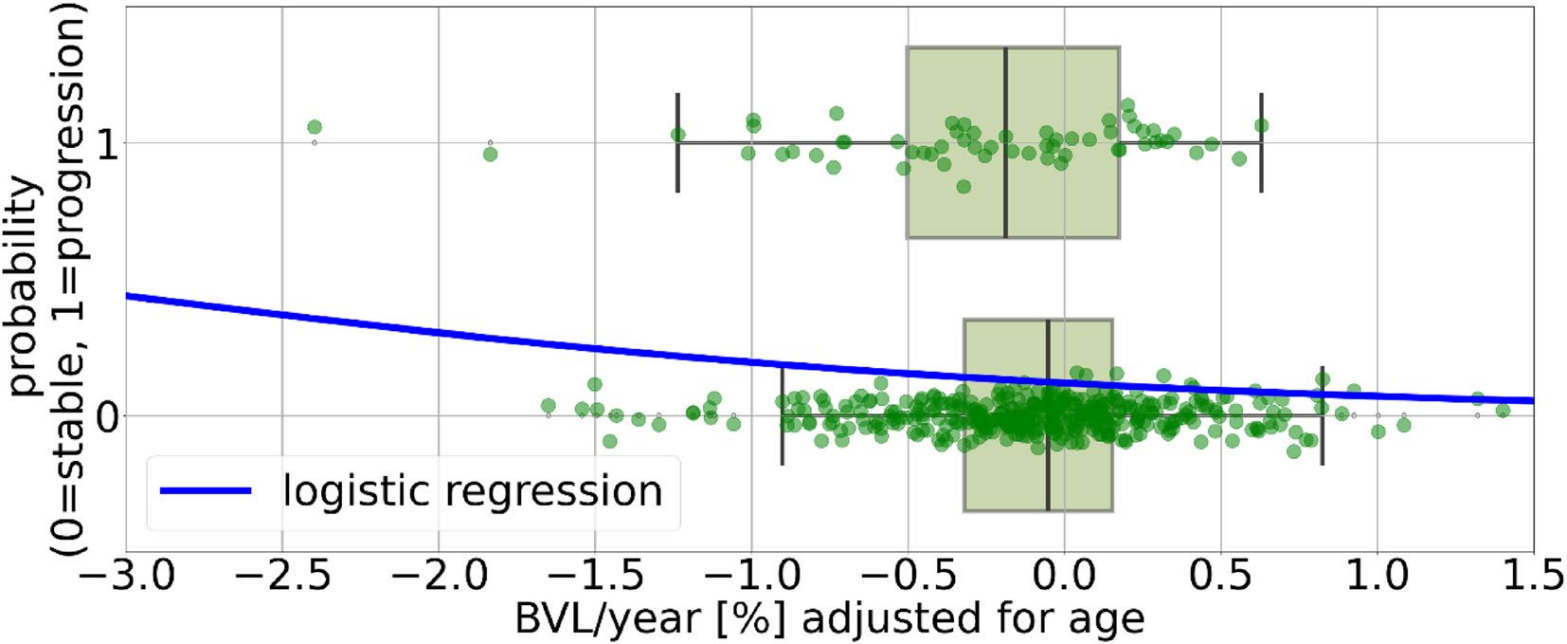
The patient cohort was dichotomized into those with EDSS progression, n = 59, 13% (according to the sensitive definition of EDSS progression as defined in the methods section) and those with stable EDSS. Patients with stable EDSS had a mean BVL/year (SD) of −0.09% (0.49) compared to −0.26% (0.56) in those with EDSS progression (*p* = 0.02).The blue line represents the logistic regression function modeling the probability of the two classes as a function of BVL/year adjusted for age. *EDSS* Expanded disability status scale, *BVL* brain volume loss

## Methods

**Fig. 2:**
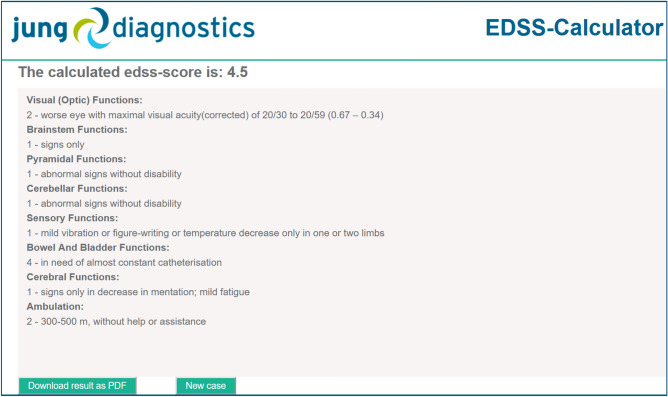
Illustration of the publicly available EDSS calculator (https://edss-calculator.com/) used in this study to collect EDSS scores in a standardized way

Study inclusion criteria are as follows: (a) diagnosis of MS based on the 2017 revised McDonald criteria, (b) relapsing clinical course, (c) age 18–55 years, (d) meeting at least one of the following disease activity criteria: at least 10 T2-lesions at the time of inclusion [43] OR at least 1 documented relapse within the past 12 months OR at least 1 Gd-enhancing lesion on brain MRI within the past 12 months OR at least 1 new/enlarged T2-lesion within the past 12 months. Each study participant is followed for 36-months comprising a baseline (BL) visit, followed by three follow-up (FU) visits at approximately 12, 24, and 36 months. The scan intervals between two consecutive scans ranged from a minimum of 10 months to a maximum of 20 months. An MRI examination is conducted at each visit. At each study visit, three types of data are collected: (**1) clinical status:** current medication, therapy changes since the last visit, and relapse activity are documented. At BL time since first MS diagnosis was additionally recorded. (**2) clinical scores**: MS-related disability is assessed using the Expanded Disability Status Scale (EDSS) and optionally the Symbol Digit Modalities Test (SDMT). Both EDSS and SDMT scores are collected in a standardized, barrier-free manner via a web-based tool (https://edss-calculator.com/). For EDSS assessment, all eight sub-scores were recorded, and the overall EDSS score is derived based on these sub-scores (see Fig. 2 of the appendix for illustration) using standard rules as described in [20]. (**3) Brain MRI examination:** each participant undergoes at least four standardized brain MRI examinations during the 36-month study period (at BL and then annually). The MRI protocol follows the 2021 MAGNIMS-CMSC-NAIMS consensus recommendations [45]. The MRI protocol included a high-resolution isotropic T1-weighted gradient echo sequence (3D MPRAGE) and a 2D or 3D T2-FLAIR sequence. Patients are required to undergo FU scans on the same MRI scanner, using the same protocol settings to ensure consistency and comparability across time points.

All procedures are integrated into routine clinical practice (standard-of-care) and no additional data collection is required, in line with the non-interventional nature of the study. Patient data pseudonymization was implemented to warrant data protection in accordance with the General Data Protection Regulation (EU 2016/679). Clinical data and study related information are recorded at study sites after each visit and entered into an electronic case report form via a secure web portal (https://ms-cockpit.de). The study was approved by local ethics committees of the participating centers. Patients had given written informed consent.

### MRI data analysis

All MRI data sets are transferred to jung diagnostics GmbH for central data analysis. For this analysis the following parameters were computed using the proprietary processing pipeline developed by jung diagnostics GmbH (Biometrica; version 1.1):

**T2-lesions load a BL**: T2-lesions count and load (in ml) were measured at each time point based on the 2D or 3D FLAIR image and the 3D MPRAGE image as confirmatory sequence [14] deploying a fully automated approach based on convolutional neural networks (CNN) [19].

**Whole brain volume at BL:** For each time point whole brain volume was automatically computed based on the BL 3D MPRAGE image using an inhouse developed and previously described and validated CNN model [30]. These volumes were then normalized to z-scores using a normative database of 5,059 healthy controls, with adjustments for age and total intracranial volume (TIV), as described in [29].

**Lesion activity:** New or enlarged (new/enlarged) T2-lesions between BL and FU were segmented fully automatically using another inhouse developed CNN model [18]. Each automatically detected new/enlarged T2-lesions was manually checked by an experienced rater and corrected if necessary. The total number of new/enlarged was normalized to the time interval between BL and FU scan.

**Whole brain volume loss (BVL):** Recently, “BrainLossNet”, a deep learning-based approach for estimating percentage BVL from longitudinal MRI, was proposed and validated [27, 34]. This novel approach provides BVL estimates which are in good agreement with the classical SIENA method [40] while offering significantly greater robustness [27]. Furthermore, BrainLossNet demonstrated a higher effect size for detecting accelerated BVL and for distinguishing MS patients with and without disability progression, outperforming SIENA-based BVL estimations [34]. For each subject, BrainLossNet was used to estimate the percentage BVL between two time points. The BVL rate (BVL/year) was then determined by dividing the percentage BVL by the time interval (in years) between the BL and FU scan. It is well known that BVL/year depends on the age of patients [31, 38]. The BVL/year was therefore adjusted for age by using a longitudinal normative database comprising 563 healthy individuals not suffering from an ongoing neurological or psychiatric condition and without structural abnormalities in the brain [34]. The BVL/year adjusted for age quantitatively characterizes the extent to which an individual BVL rate deviates from BVL in healthy individuals with the same age. Positive residuals indicate slower than expected BVL rates, negative values indicate accelerated BVL. A BVL/year (adjusted for age) threshold of $-$0.4% can be considered as the threshold which distinguishes BVL due to normal aging from pathological BVL [11, 31, 34].

The analysis in this study is based on data from the BL and the first FU scan.

**Table 1:** Patient characteristics of *n* = 453 RRMS patients used for the data analysis in this study

	25%/50%/75%
Interval	1.0/1.0/1.2
age at BL	34.4/40.4/48.5
time since first MS diagnosis at BL	2.1/5.7/11.4
EDSS at BL	1.5/2.0/2.5
ΔEDSS	0.0/0.0/0.0
T2-lesion vol at BL	1.2/3.1/8.3
T2-lesion no. at BL	12.0/22.0/34.0
z-score BPV at BL	$-$1.7/$-$0.9/$-$0.2
no. new/enlarged T2-lesions per year	0.0/0.0/0.6
BVL/year	$-$0.5/$-$0.3/$-$0.0
BVL/year adjusted for age	$-$0.4/$-$0.1/0.2

*BL* baseline, *EDSS* Expanded disability status scale, *ΔEDSS* difference between follow-up and baseline EDSS*, BPV* brain parenchymal volume, *BVL* brain volume loss

**Table 2:** Percentage of *n* = 453 RRMS patients with specific characteristics

	%
Without relapse	75.5
Without new/enlarged T2-lesions	72.2
Without new/enlarged T2-lesions and without relapse	56.3
ΔEDSS < 0/ΔEDSS = 0/ΔEDSS > 0	16.3/69.1/14.6
Normal BVL/year adjusted for age	77.3

*EDSS* Expanded disability status scale, *ΔEDSS* difference between follow-up and baseline EDSS, *BVL* brain volume loss

**Table 3:** Pearson’s correlation coefficients with p-values between disability-related scores and MRI-derived metrics

		T2-lesion no. at BL	z-value BPV at BL		Age at BL	EDSS at BL	Time to first MS diagnosis	T2-lesionvol at BL	T2-lesion no. at BL	z-value BPV at BL	No. new/enlarged lesions per year	BVL/year adjusted for age
All patients (*n* = 453)												
EDSS at BL	**0.28** (*p* < 0.001)	**0.12** (*p* = 0.0093)	$-$**0.31** (*p* < 0.001)	ΔEDSS	0.02 (*p* = 0.6677)	$-$**0.13** (*p* = 0.0041)	0.02 (*p* = 0.7492)	0.05 (*p* = 0.312)	0.04 (*p* = 0.4481)	$-$0.02 (*p* = 0.612)	0.06 (*p* = 0.1738)	$-$**0.14** (*p* = 0.0024)
Patients without relapse (*n* = 342)												
EDSS at BL	**0.30** (*p* < 0.001)	**0.13** (*p* = 0.0204)	$-$**0.30** (*p* < 0.001)	ΔEDSS	0.04 (*p* = 0.4775)	$-$**0.13** (*p* = 0.0155)	0.05 (*p* = 0.387)	0.04 (*p* = 0.4564)	0.03 (*p* = 0.522)	0.01 (*p* = 0.9234)	0.02 (*p* = 0.755)	$-$**0.16** (*p* = 0.0031)
Patients without new/enlarged T2-lesions and without relapse (*n* = 255)												
EDSS at BL	**0.28** (*p* < 0.001)	0.08 (*p* = 0.1855)	$-$**0.33** (*p* < 0.001)	ΔEDSS	0.08 (*p* = 0.2037)	$-$**0.13** (*p* = 0.0341)	0.09 (*p *= 0.171)	0.07 (*p* = 0.2354)	0.06 (*p* = 0.3778)	$-$0.02 (*p* = 0.7257)	nan	$-$**0.17** (*p* = 0.008)

The left block of the table shows the associations between BL EDSS and MRI parameters, the right block shows associations with ΔEDSS. Statistically significant (*p* < 0.05) correlations are highlighted in bold

*BL* baseline, *EDSS* Expanded disability status scale, *ΔEDSS* difference between follow-up and baseline EDSS, *BPV* brain parenchymal volume, *BVL* brain volume loss

**Table 4:** Correlations between functional EDSS sub-scores and MRI parameters

	T2-lesion vol at BL	T2-lesion no. at BL	z-value BPV at BL		No. new/enlarged lesions per year	BVL/year adjusted for age
Visual (optic) at BL	**0.19** (*p* = 0.0021)	0.06 (*p* = 7.0239)	$-$0.10 (*p* = 1.4739)	Δvisual (optic)	0.14 (*p* = 0.1112)	$-$0.12 (*p* = 0.4076)
Brainstem at BL	0.14 (*p* = 0.0911)	0.10 (*p* = 1.0607)	$-$**0.25** (*p* < 0.001)	Δbrainstem	0.07 (*p* = 5.517)	$-$0.04 (*p* = 13.7505)
Pyramidal at BL	**0.29** (*p* < 0.001)	0.14 (*p* = 0.1025)	$-$**0.31** (*p* < 0.001)	Δpyramidal	$-$0.02 (*p* = 26.3983)	$-$0.09 (*p* = 2.4165)
Cerebellar at BL	**0.28** (*p* < 0.001)	0.21 (*p* < 0.001)	$-$**0.36** (*p* < 0.001)	Δcerebellar	$-$0.00 (*p* = 38.4715)	$-$0.15 (*p* = 0.0563)
Sensory at BL	0.04 (*p* = 0.2376)	0.03 (*p* = 23.2692)	$-$0.09 (*p* = 2.6583)	Δsensory	0.04 (*p* = 14.6012)	$-$0.08 (*p* = 4.3452)
Bowel/bladder at BL	**0.15** (*p* = 0.0406)	$-$0.01 (*p* = 35.0321)	$-$**0.16** (*p* = 0.0259)	Δbowel/bladder	$-$0.05 (*p* = 13.53)	0.03 (*p* = 23.2468)
Cerebral at BL	**0.18** (*p* = 0.0036)	0.10 (*p* = 1.6365)	$-$**0.26** (*p* < 0.001)	Δcerebral	0.01 (*p* = 35.7291)	$-$**0.19** (*p* = 0.0027)
Ambulation at BL	**0.24** (*p* < 0.001)	0.03 (*p* = 23.5332)	$-$**0.21** (*p* < 0.001)	Δambulation	$-$0.00 (*p* = 39.9316)	$-$**0.17** (*p* = 0.0111)

The *p*-values are multiplied by the number of tests (40) to account for multiple testing (Bonferroni correction). Statistically significant (*p* < 0.05) correlations are highlighted in bold

*BL* baseline, *Δ* difference between follow-up and baseline, *BPV* brain parenchymal volume, *BVL* brain volume loss

### Statistical analysis

To assess changes in disability over time the ΔEDSS was calculated as the difference between FU and BL scores, with positive values indicating an increase in disability. To explore potential associations with ΔEDSS, we first tested the following BL parameters for Pearson correlation and statistical significance: time since first MS diagnosis (in years), EDSS, patient age (in years), T2-lesion load (volume in mL), number of T2 lesions, and brain parenchymal volume z-score (z-score BPV). The following longitudinal parameters were also evaluated: BVL/year (adjusted for age) and the number of new or enlarged T2 lesions. Parameters that showed a statistically significant association with ΔEDSS were subsequently included as fixed effects in a linear mixed-effects model. Scanner type (see Table 5 in the appendix) was included as a random intercept to account for inter-scanner variability. Collinearity among independent variables was assessed using the variance inflation factor (VIF).

As described above, the EDSS score was derived from eight functional sub-scores. As an experimental analysis we computed Pearson correlation coefficient between individual functional EDSS scores and the MRI derived parameters and tested them for statistical significance. The p-values were adjusted for multiple testing by multiplying the original *p*-value by the total number of performed tests (Bonferroni correction).

Patients were dichotomized into two groups: those with EDSS progression and those with stable EDSS. EDSS progression was defined as an increase of 1.5 points from a BL EDSS score of 0, 1.0 point for BL scores between 1.0 and 5.5, and 0.5 points for scores above 5.5 [24]*.* Given the short observation interval, substantial EDSS increases were expected to be rare. Therefore, a second, more sensitive definition of EDSS progression was also applied: an increase of 1.5 points from a BL score of 0, or an increase of 0.5 points for all BL scores greater than 0.

A logistic regression model was used to assess the association between BVL/year, adjusted for age, and EDSS progression. The model included BVL/year (age-adjusted) and a constant as independent variables. Additionally, BVL/year (adjusted for age) was compared between the two groups using a t-test to evaluate statistical significance. A Kolmogorov–Smirnov test and visual inspection of the distribution were performed to assess whether BVL/year (adjusted for age) follows a normal distribution.

## Results

The main results are shown in Figure 1 and in Tables 1, 2, 3, and 4. Data collection started in July 2022 (first patient first visit) and to date a total of 1,331 active relapsing–remitting MS patients have been enrolled. As the study is still ongoing, FU scans are not yet available for all participants. As of now, 459 patients have undergone the one-year MRI FU scan, along with corresponding clinical FU data. Data sets from six patients were excluded due to significant image artifacts or because BL and FU scans were acquired on different MRI scanners, which would compromise comparability and introduce potential errors. The remaining 453 patients (71% female) were managed in 18 participating MS centers. Table 5 of the appendix provides information about the MRI scanner models and protocols used. Table 6 of the appendix shows the distribution of the therapies. Out of the 453 patients 80 patients (17%) switched therapy during the observation period. Table 1 presents a description of the enrolled patients. As shown in Table 2, 75% of the patients did not experience any relapses during the observation period, and 56% had neither relapses nor new/enlarged T2-lesions. Among the 453 patients, 69% showed no change in their EDSS score, 16% showed an improvement in EDSS and 14% worsening in EDSS. 77% had a normal age-appropriate BVL/year value. Table 3 presents the correlation coefficients between disability scores and MRI metrics. Analysis was performed for all 453 patients and then repeated for all patients without relapses during the observation period (*n *= 342) and for all patients without relapses and without new/enlarged T2-lesions (*n* = 255). BL EDSS scores showed a moderate positive correlation with T2-lesion volume (r ≈ 0.3) and a negative correlation with z-scores of brain volumes (*r* = $-$0.30 to $-$0.33). ΔEDSS was significantly correlated only with BL EDSS and BVL/year, as shown in Table 3. Similar patterns of correlation were observed in the sub-cohort of patients without clinical relapses (*n* = 342), as well as in the group of patients without new or enlarged T2 lesions and without relapses (*n* = 255). A linear mixed-effects model was then fitted with scanner as a random intercept and BVL/year (age-adjusted) and BL EDSS as fixed effects. Scanner with fewer than 5 patients were excluded from the mixed-effects analysis, to reduce instability due to low group sizes. The final model included 428 patients across 11 scanner with each scanner represented by at least 12 patients. The mixed-effects model converged successfully and revealed a significant negative association between BVL/year and ΔEDSS (*β* = –0.104, *p* = 0.020), as well as a significant effect of BL EDSS (*β* = –0.038, *p* = 0.029). Although minor scanner variability was present (random intercept variance = 0.014) indicating that the observed association is robust and not driven by scanner-related measurement differences. The variance inflation factor (VIF) was 1.004 for both BL EDSS and BVL/year, indicating no multicollinearity. Additionally, no meaningful correlation was observed between BL EDSS and BVL/year (r = $-$0.01).

Table 4 presents the correlations between functional EDSS sub-scores and MRI parameters. Among the functional changes, only Δcerebral and Δambulation scores were significantly correlated (after adjustment for multiple testing) with BVL/year ($-$0.19 and $-$0.17, *p* < 0.005).

**Fig. 3:**
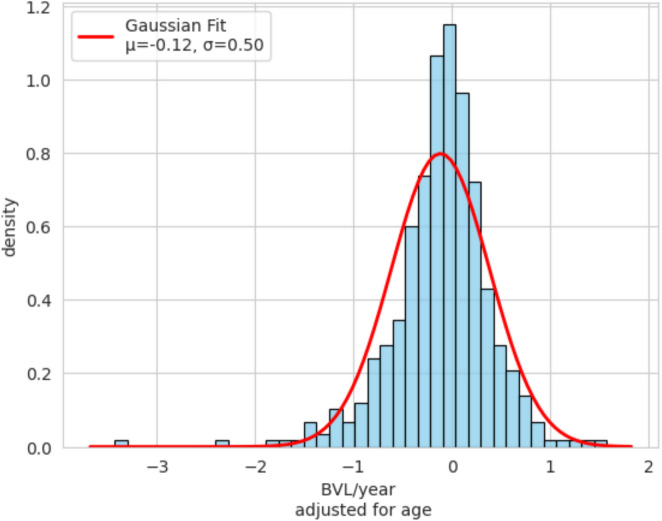
Distribution of brain volume loss (BVL)/year adjusted for age

Patients were dichotomized into those with EDSS progression (*n* = 20, 4.4%) and those with stable EDSS (*n* = 433) based on the standard definition of EDSS progression (see Methods section for exact criteria). Patients with stable EDSS had a mean (SD) BVL/year of $-$0.10% (0.49), whereas those with EDSS progression showed a significantly greater loss at $-$0.35% (0.73) (*p* = 0.03). While formal testing of BVL/year variable showed a statistically significant deviation for normality (Kolmogorov–Smirnov test, *p* = 0.007) visual inspection suggested (see distribution in Fig. 3 of the appendix) the data was approximately normally distributed, supporting the use of the parametric t-test. Using the more sensitive definition of EDSS progression, 59 patients (13%) met the criteria for progression. Under this definition, patients with stable EDSS had a mean BVL/year of $-$0.09% (0.49) compared to $-$0.26% (0.56) in those with EDSS progression (*p* = 0.02). For both definitions, BVL/year was significantly associated with EDSS progression in the logistic regression model (*p* < 0.05). According to the model, patients with an age-adjusted BVL/year of $-$0.5% had an estimated 5% probability of EDSS progression using the standard definition. A decrease in BVL/year from $-$0.5% to $-$1.0% increased the risk from 5% to 7%, representing a 40% relative increase. Using the more sensitive definition, a BVL/year of $-$0.5% corresponded to a 15% probability of EDSS progression. A further reduction BVL/year to $-$1.0% increased this risk to 19%, representing a 26% relative increase. A box plot illustrating this association, along with the logistic regression model results for the more sensitive definition, is presented in Fig. 1.

The analysis was repeated excluding the 80 patients who switched therapy during the observation period. The correlation remained nearly unchanged, differing by no more than one decimal place.

## Discussion

After a mean observation period of 1.2 years (median 1.0 year), we found weak but robust and statistically significant associations between ΔEDSS and BVL/year. There are multiple reasons for the limited strength of the associations. First, the EDSS is a relatively coarse measure of disease progression, with known limitations. The EDSS heavily weighs walking ability, making it less sensitive to upper limb dysfunction, cognitive impairment, and other MS symptoms [4]. The EDSS has a lack of granularity since the final EDSS score collapses functional impairments into a single value, masking variability in different functional domains [22]. In addition the EDSS is known to have high inter-rater variability [6]. As described above, the EDSS values were collected using a web-based application and the final EDSS score was automatically calculated from the 8 EDSS sub-scores. The use of electronic input forms has been shown to improve scoring consistency and reduce variability [8].

Another reason for the weak association is that disease progression is multifactorial and may not be fully captured by MRI-based biomarkers of the brain (spinal cord is for instance is not considered in this study). However, our results suggest that BVL/year is clinically relevant (despite the week association). The logistic regression model revealed a 40% relative risk of EDSS progression increase in patients with strong brain atrophy. That means a patient with a BVL/year of $-$1.0% has a 40% higher risk of being in the group of EDSS progressors than a patient with only $-$0.5%.

We also found a negative correlation between BL EDSS and ΔEDSS which means that patients with a lower EDSS at BL tend to show greater increases in EDSS over time. This is clinically plausible and supported by existing literature [36]. However, BVL/year and EDSS at BL seem to be independently associated with ΔEDSS and no collinearity was observed.

Our findings suggest that accelerated brain atrophy is linked to an increased risk of clinical progression. This correlation was also observed in a subset of patients who experienced no relapses, as well as in those without relapses and without any new or enlarged T2 lesions, suggesting that BVL is relevant for disability progression even in the absence of inflammatory disease activity. Moreover, the fact that this association could be detected within a relatively short observation period highlights the potential of BVL as a valuable marker for PIRA, both in clinical trials and routine patient care.

A recent literature review on the association between BVL and disability progression in MS revealed a total of 36 relevant studies published since 2010 [23]. However, most studies showing a significant association have a follow-up period of 5 to 10 years [23]. Out of the 36 studies only 7 studies reported a study duration of less than 2 years and from these 7 studies only 4 reported statistically significant associations. From the remaining four studies [3, 9, 10, 16], three studies [9, 10, 16] reported significant correlations between EDSS at BL and BVL/year. However, EDSS at BL does not predict the change of disability during the observation period. Among the 36 publications identified in the mentioned review article, only one single study [3] had an observation period of less than two years and reported a significant correlation between non-annualized BVL and changes in EDSS. This monocentric study, conducted in a similar but much smaller cohort of 102 relapsing–remitting MS patients, had a mean follow-up interval of 1.05 years and reported a correlation coefficient of *r* = −0.148 with a significance level of *p* = 0.041. However, the authors noted that this significance disappeared after annualizing BVL. Since both non-annualized BVL and disability progression tend to increase with the duration of the observation period, using non-annualized BVL may be misleading, because the observation period itself could introduce a hidden correlation between non-annualized BVL and EDSS change, potentially confounding the results. In summary, to our knowledge the results presented in this work demonstrate for the first time a robust and significant correlation between the change in EDSS and BVL/year with an observation time of less than two years.

Quantifying BVL is inherently challenging due to image noise and measurement variability. This may explain why conventional gold-standard methods such as SIENA and Jacobian integration have struggled to demonstrate robust associations within short observation periods. To address these limitations, we applied a recently introduced deep learning–based method (BrainLossNet) [27, 34], designed to enhance the robustness and sensitivity of longitudinal brain volume measurements.

As already mentioned, currently available DMT reduce the risk of relapses and lesion activity significantly but have only limited impact on PIRA [17, 21]. While relapses and lesion activity have historically guided treatment decisions, it is now clear that a significant portion of disease progression occurs independently of acute inflammation [17]. This highlights the need for reliable biomarkers that can detect, monitor, and predict PIRA. Several candidates have recently been proposed [5]. Among them, serum neurofilament light chain (sNfL) is gaining attention as a promising marker for silent disability progression [7].

This study has several limitations that should be acknowledged. According to the original definition of PIRA EDSS progression must be confirmed after 3 or 6 months. However, due to the study design, it was not feasible to collect clinical data between MRI scans. In addition, the mean observation period in the current dataset is only 1.2 years, which has not yet allowed for such confirmation. The study is ongoing and additional EDSS data points will be collected when the second and third MRI are acquired.

As mentioned in the methods section, RRMS patients were included if they showed some disease activity in the 12 months prior to inclusion and if there was no intention to switch therapy. However, the exact history of relapses and treatment prior to inclusion may vary between patients. This potentially could influence the result.

The study reported correlations, which do not imply causality and do not permit conclusions about the predictive value of BVL for future disability. Although our results suggest an association between BVL and disability progression, a definitive analysis of predictive power requires additional FU examinations. We plan to extend the analysis with future FU data to evaluate whether BVL can serve as a reliable predictor of disability progression. Second, our current analysis focuses exclusively on BVL of the whole brain. Previous research suggests that regional atrophy, particularly thalamic volume loss, may be a more sensitive marker of neurodegeneration and clinical progression [13, 28, 35]. However, existing methods for quantifying thalamic volume loss are usually based on Jacobian determinant approaches which tend to high noise levels in particularly when evaluated regionally or used for short observation periods [28]. Currently, we are extending the DL-based framework “BrainLossNet” [27, 34] for subregions such as the thalamus. It will be an interesting future task to extend the study also using regional atrophy measures.

We chose to use time since MS diagnosis as a proxy for disease duration. While time since first symptoms would be a more accurate reflection of disease duration, this measure also has limitations, as it relies on patient recall and the initial symptoms are often ambiguous or misattributed. In contrast, time since diagnosis is typically well-documented in medical records and can be reliably extracted. Therefore, we chose to use it as a practical and consistent proxy for disease duration.

In conclusion, BVL/year, adjusted for age, was robustly and significantly associated with progression of disability, even without the presence of relapses or active lesions. BVL/year may serve as a risk factor for short-term progression of disability in multiple sclerosis.

## Appendix

**See **Figs. 2 and 3

See Tables (5 and 6)

**Table 5:** Scanner models and MRI protocol settings for 453 MS patients included into this study

No. of patients per scanner	Scanner			T1 gradient echo sequence					Flair				
	Manufacturer	Model	Field strength	TR	TE	Flip angle	Slice thickness	Pixel spacing	TR	TE	Flip angle	Slice thickness	Pixel spacing
123	SIEMENS	Skyra	3	2300.0	3.0	9	1.0	1.0	5000	393	120	1.0	1.0
107	SIEMENS	Avanto	1.5	1900.0	2.8	8	0.9	1.0	9000	137	180	5.0	0.7
40	SIEMENS	MAGNETOM Skyra	3	1400.0	2.3	8	1.0	0.9	5000	395	120	1.0	0.5
37	SIEMENS	Avanto	1.5	980.0	3.0	15	1.0	1.0	11,450	111	150	3.0	0.5
36	Philips	Ingenia	1.5	7.5	3.4	8	1.0	0.6	4800	357	90	1.2	1.0
22	Philips	Ingenia	1.5	7.5	3.5	8	1.1	0.6	4800	300	90	1.2	0.6
20	SIEMENS	MAGNETOM Skyra	3	1800.0	3.6	8	1.0	1.1	9000	105	150	3.0	0.8
14	SIEMENS	MAGNETOM Skyra	3	2200.0	2.5	8	0.9	0.9	5000	376	120	0.9	0.5
14	SIEMENS	MAGNETOM Skyra	3	1400.0	2.6	15	1.0	0.4	7000	379	120	1.0	0.5
12	SIEMENS	MAGNETOM Skyra	3	2200.0	2.5	8	0.9	0.9	5000	376	120	0.9	0.5
12	SIEMENS	Avanto	1.5	1560.0	2.8	8	1.0	1.0	8010	89	150	3.0	0.4
4	SIEMENS	MAGNETOM Aera	1.5	2200.0	3.2	8	1.0	1.0	9000	82	150	4.0	0.7
4	Philips	Ingenia	1.5	7.7	3.5	8	1.1	0.6	4800	300	90	1.2	0.6
2	SIEMENS	MAGNETOM Skyra	3	1400.0	2.6	15	1.0	0.4	7000	379	120	1.0	0.5
2	Hitachi	ECHELON_Smart	1.5	570.0	8.0	90	1.0	0.5	8000	120	90	5.0	0.4
2	SIEMENS	Avanto	1.5	1560.0	2.8	8	1.0	1.0	8010	89	150	3.0	0.4
1	SIEMENS	MAGNETOM Altea	1.5	1700.0	2.9	8	1.0	1.0	5830	76	180	5.0	0.4
1	GE	Signa HDxt	1.5	8.1	3.4	12	1.6	1.0	10,002	138	90	5.0	0.5

**Table 6:** Distribution of the *n* = 453 MS therapies at BL

Therapy at BL	*n*
Dimethyl fumarate	71
Ocrelizumab	61
Glatiramer acetate	43
Teriflunomide	38
Ozanimod	38
Fingolimod	33
No therapy	32
Natalizumab	28
Interferon beta	28
Cladribine	24
Ofatumumab	23
Diroximel fumarate	14
Unknown	10
Alemtuzumab	5
Ponesimod	4
Rituximab	1
